# Dynamic electro-regulation of the stiffness gradient hydrogels†

**DOI:** 10.1039/c7ra11382j

**Published:** 2018-02-09

**Authors:** Runhuai Yang, Haiyi Liang

**Affiliations:** Department of Biomedical Engineering, School of Life Science, Anhui Medical University 230032 Hefei China; CAS Key Laboratory of Mechanical Behavior and Design of Materials, University of Science and Technology of China 230027 Hefei China hyliang@ustc.edu.cn; IAT-Chungu Joint Laboratory for Additive Manufacturing, Institute of Advanced Technology, University of Science and Technology of China Hefei 230026 Anhui China

## Abstract

Hydrogels are promising biomaterials which provide bionic environments to study the effect of stiffness. A hydrogel with dynamically changeable stiffness can be used to further understand the dynamic biological processes such as embryonic development, tumorigenesis, *etc.* here we present an electro-regulation method which can dynamically control the stiffness of hydrogels with ionic crosslinks. By applying an electric field and controlling the voltage applied to the hydrogel the mechanical properties of the hydrogel can be changed. The voltage can directly change the stress–relaxation properties of the hydrogel, and after the voltage was applied for hundreds of seconds, a gradient stiffness was generated along the direction of the electric field. The value of gradient stiffness can be regulated by charge. With the increase of charge, the stiffness of the hydrogel near the anode increases, while the stiffness of the hydrogel near the cathode decreases. Experiments show that the electro-regulation method allows dynamic manipulation of the material properties of the hydrogel with ionic crosslinks. Dynamic electro-regulation of hydrogels is a powerful tool that will promote the study of the dynamic microenvironments and lead to a better understanding of the fundamental phenomena of the extracellular matrix.

## Abstract 2

A reversible electro-regulation process was developed which can dynamically regulate the stiffness gradient of hydrogels (10 to 30 kPa).

## Graphical abstract

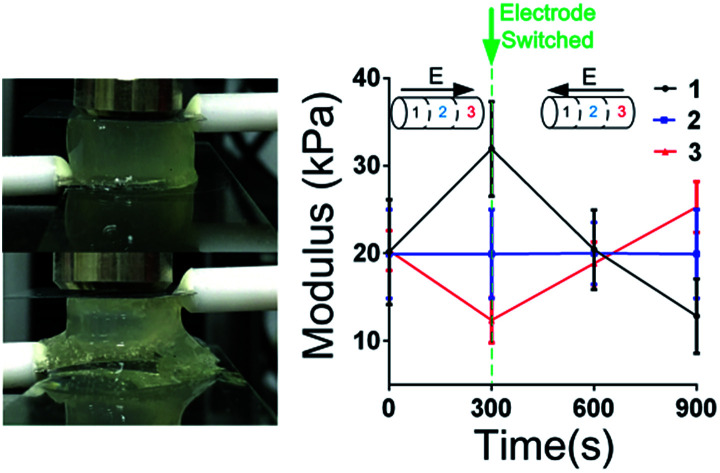

Hydrogels are network polymeric materials with high water contents. Due to their biocompatibility and biodegradability, hydrogels are considered as promising materials which can mimic the native extracellular matrix.^1,2^ Current research of hydrogels shows that they are promising materials for tissue scaffolds, cell encapsulation and bioinks of 3D bioprinting.^3^ The mechanical properties of hydrogels are a critical factor, especially when hydrogels are used as an extracellular matrix (ECM) or tissue scaffold.^4^ Studies proved that many kinds of cell behaviours, such as adhesion, proliferation, migration, and differentiation are dependent on the stiffness of the substrate. For example, it is reported that a substrate with nearly 30 kPa stiffness can induce osteogenic differentiation of mesenchymal stem cells (MSCs) while a softer substrate can enhance myogenic and neurogenic differentiation.^5^ Therefore, many researchers have focused on the control of the stiffness of the culture environment.

Most studies were based on the substrate with certain modulus or with constant mechanical properties throughout the culture period.^6^ However, it is known that the biological processes are dynamic, and the stiffness of the ECM is with gradient properties and changeable.^7,8^ The gradient properties of stiffness commonly exist in the extracellular environment.^9^ Natural stiffness gradients occur in both pathological process and physiological process. For example, the myocardial infarction can induce the environment to have a gradient of nearly 8.7 kPa mm^−1^, and myocardium can induce the environment to have a gradient of nearly 0.6 kPa mm^−1^.^10^ Besides, studies on the mechanical properties of the ECM reveals that microenvironment dynamically change its matrix stiffness and significantly influence the biological processes such as embryonic development, healing and tumorigenesis.^11^ Dynamic changes in ECM stiffness can provoke changes in cell behaviour.^12^ For example, the time of ECM stiffening could induce the human mesenchymal stem cells into adipocytes or osteogenic.^8^ To mimic the formation of adult myocardium, the continuous increase of tissue stiffness of hydrogels also makes the pre-cardiac cells form more muscle fibres.^13^

Methods to control the hydrogels with gradient and dynamic stiffness are important to mimic the natural ECM environment. However, most methods initially controlled the mechanical property of hydrogels by changing polymer concentration or chain length, and the mechanical properties kept constant throughout the culture period. Degradation offers a means to soften gels over time. Degradable linkers have been utilized for controlled degradation and the hydrogels became collapse.^14^ Some tunable hydrogels were made to degrade hydrolytically,^15^ and later engineered with degradation sites tailored to specific proteases.^16^ However, these methods mostly were in a passive and uncontrolled manner, and it is not easy to realize both dynamic soften (low to ∼10 kPa) and stiffen (up to ∼30 kPa).^17^

Thus, the main aim of the present study was to develop (i) a substrate with a well-defined stiffness gradient along the designated direction using a simple technique; and (ii) a dynamic tuning method to time-dependant control the stiffness of the gradient hydrogels. Alginate hydrogel is adopted in our system as model material because its high biocompatibility and it can easily be molded by addition of divalent cation. The stiffness of alginate hydrogel can be controlled by the calcium concentration, and can be regulated/modulated by moving the calcium ions. Based on these properties, an electro-regulation method is proposed to control the stiffness gradient for the alginate hydrogel. The hydrogel can be softened or stiffened. Besides, this method has the ability to time-dependently control the trend of softening or stiffening. The speed of softening or stiffening can be controlled by the voltage, and the value of softening or stiffening can be precisely controlled by controlling the conduction time. Finally, the application of electro-regulation allows for dynamic control of the hydrogel stiffness and generates precise gradient hydrogels.

To reduce the individual difference of hydrogels after gel formation, ethylenediaminetetraacetic acid calcium disodium salt hydrate and d-(+)-gluconic acid delta-lactone (Ca-EDTA/GDL) crosslinked alginate was prepared. This slow-releasing alginate hydrogel can slowly release calcium ions and the hydrogel spent 10 hours to reach a stable and homogeneous stiffness. After that, the stress–relaxation behaviours of alginate hydrogel with or without electric filed were observed.

The stress–relaxation behaviour provides information of the dynamic stress with a constant strain applied. By using the universal testing machine, electrochemical workstation and platinum electrode, the stress–relaxation curves with different voltage were obtained. A cylinder-shaped gel was placed between two impermeable rigid plates and the electrode was positioned on the both sides of the hydrogel. A strain quickly applied to the gel while the force applied to the sample was 4.5 kPa. The voltage was applied after the strain was applied to the gel. Subsequently, the strain is held constant, and the stress was recorded as a function of time. The hydrogels applied with different voltage exhibited different stress–relaxation behaviours, as shown in Fig. 1(e). In the beginning, the pre-stress (4.5 kPa) was applied on the hydrogels. The black curve shows the control experiment while the voltage was not applied. As a viscoelastic material, the stress will decay and reach a constant nonzero value. Without the effect of the electric, the mechanical property of hydrogel was stable and the value of the whole course was higher than 4 kPa. The other three curves present the alginate hydrogels with different voltages (1.5 V, 3 V, 4.5 V). After the voltage was turned on, the stress was significantly reduced, showing that the structure of the hydrogel was changed. It is obviously that with the increase of the voltage, the speed of decay increases. The rate of decay was related to the electric current. For the sample applied by 1.5 V, the stress only decreased by ∼10% in 350 s. For the sample applied by 3 V, the hydrogel relaxed half of the stress in 350 s. The load in the gel which was applied 4.5 V took ∼350 s for the decay to nearly zero, presenting that the part of the hydrogels exhibit viscoelastic liquid properties.

**Fig. 1:**
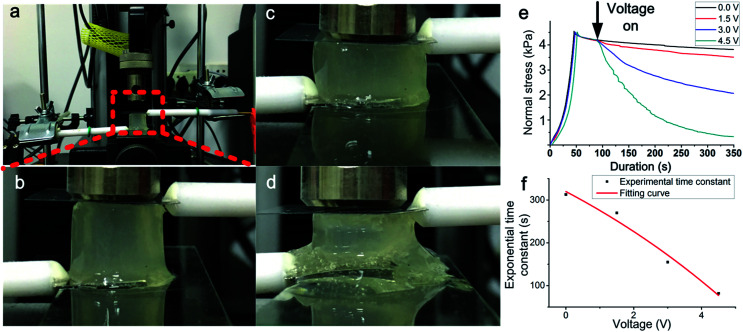
The schematic of the stress–relaxation test of the alginate hydrogel while the voltage was applied. The pre-stress was applied by the force sensor of the stretcher. (a) Photo shows the experimental setup. (b) Zoom in photo shows the setup of the sensor, electrode and hydrogel samples. (c) The pre-stress was applied to the hydrogels and the hydrogel was compressed. (d) After the voltage was applied by the electrode, the structure of the hydrogel was disrupted and the hydrogel near cathode was changed to liquid. (e) The stress–relaxation curves of the hydrogel for each voltage value. (f) The influence of the voltage on the exponential time constant (*τ*) of the stress–relaxation curves.

An exponential decay equation was used to fit the curve after the voltage applied on the sample. The *τ* (exponential time constant) of each curve was shown in Fig. 1(f)*τ* shows that the curve induced by a higher voltage decreased faster than the curve induced by a lower voltage. With the increase of voltage, *τ* decreased more sharply. The result indicates that the voltage applied to the hydrogels can directly change the mechanical properties of the hydrogels, and voltage value and durations are both critical to the change of mechanical properties. By change the applied voltage, the speed of the change of the mechanical properties can be controlled. The results indicated that the decay speed of the loading curve can be controlled by the voltage.

Aimed at studying the gradient distribution of the hydrogels, each sample was cut into three sections after the electric field treatment (3 V, 400 s) as shown in Fig. 2. The section near the anode was laboured by the black character 1, the section near the cathode was laboured by the red character 3 and the middle one was laboured by blue 2. After the section was cut, the strain–stress test was performed on each section. The results of the strain–stress test were shown in Fig. 2(b). Each section was performed separately. The load and unload curve was both obtained. The slope of each curve reveals the stiffness of the materials. It is clearly that the section 1 quickly obtained 2.2 kPa while the strain was only 10%, and the section 3 took 30% strain to obtain a same stress. The slope of the strain–stress curve for each section is decreased along with the electric field, from section 1 to section 3. The modulus of each section was calculated by the slope of strain–stress curve and a statistic analysis for three sections was performed and was shown in Fig. 2(c). From the results it is obvious that the section 1 has the highest modulus, showing that the hydrogel near anode has a hardest stiffness. Section 3 has the lowest modulus, showing that the structure of hydrogel near cathode was changed and the stiffness become soft. The results indicated that a hydrogel with gradient stiffness was obtained by the electric field, and the hydrogel can be stiffen or soften. The section of hydrogel near the anode was stiffen, and the section of hydrogel near the cathode was soften. Therefore, a hydrogel with gradient stiffness was generated.

**Fig. 2:**
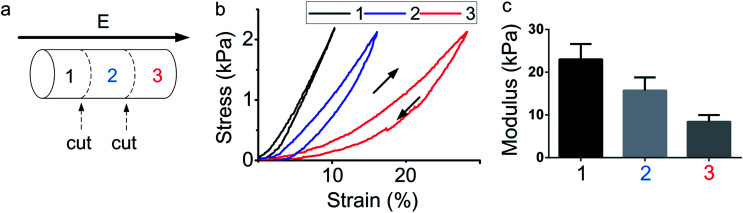
Study of the gradient property of electro-regulated hydrogel. (a) Illustration of each section (1–3) of hydrogels and (b) compressive stress–strain curves and (c) compressive modulus of the hydrogel sections along the longitudinal direction (*n* = 5, mean ± SD). The modulus of the hydrogel sections gradually decreased along the longitudinal direction with the electric field from anode to cathode, indicating the gradient formation in terms of the stiffness in the hydrogel.

The first goal of the present work is to generate a gradient stiffness, and the second goal is that the stiffness can be dynamically controlled. Therefore, the current and electric charge which is related to time was considered here. While the voltage was applied to the alginate hydrogel, a current was formed between the electrodes. The value of the current is recorded as shown in Fig. 3(a). An increase in charge was provided by the current. By integration of the current, the value of charge quantity was calculated as shown in Fig. 3(a). The charge quantity was kept increasing when the voltage is applied. The current through the hydrogels is recorded by electrochemical workstation, as shown in Fig. 3(a) by the red curve. A DC voltage (4.5 V) was applied to the hydrogels, and an electric current through the hydrogel was generated. The current decreased continuously but becomes flatter. Therefore, the charge increased continuously. The charge of the hydrogels cause the hydrogel structure was fragment and changed to liquid.

**Fig. 3:**
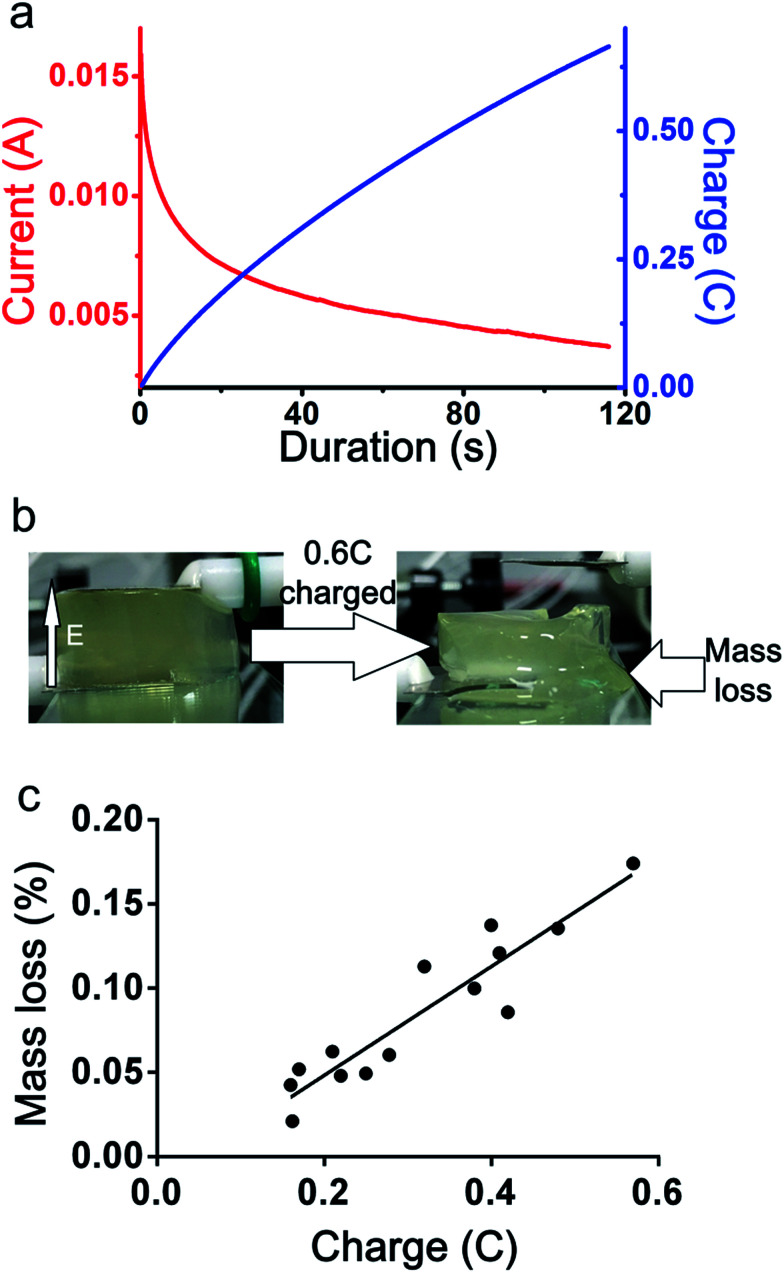
The influence of electric charge on the mass loss of hydrogel. (a) The recording of current as a function of time (red curve) and the charge which is the integration of the current (blue curve). (b) Images showing that the electric field direction and the mass loss. (c) The experimental data of the mass loss to the electric charge.

The charge indicates the movement of the ions in hydrogels, and the changed structure of the hydrogel could be indicated by the mass loss. The mass loss induced by electric charge mostly due to hydrogel collapse. More ionic bond was broken, more mass loss was observed. An experiment to plot of the weight of the mass loss *versus* the charge was performed. By recording the influence of the charge on mass loss, as shown in Fig. 3(c), the influence of the electric field on the hydrogel structure can be observed. With the electric field kept applied and the electric charge increased, the mass loss also increased. While the electric field was applied to the hydrogels, bubble was observed in the region close to cathode, which was considered to be the generation of hydrogen gas. The pH value near the cathode increased and white precipitation appeared on the cathode, which was considered to be calcium hydroxide. The lower concentration of calcium ion led to the crosslink density decreased, so that the hydrogel near the cathode became soft. In the region near anode, the mobile cation (mainly consisted of sodium ion) was forced to migrate towards the cathode under the action of electric force. The water was migrate with the movement of mobile cation, therefore, the water content decreased in the region near anode. This resulted in the increase of the stiffness near the anode.

Aimed at further studying the structure change induced by charge, the compression modulus for each section was recorded with different charge values (0C, 0.2C, 0.4C and 0.6C). Same as previous mentioned, 1 presents the section near the anode, 2 is the middle section and 3 present the section near the cathode. The results are shown in Fig. 4. The modulus of 1, 2 and 3 has no significant difference when the charge equals 0. After the voltage was applied, the modulus of section 1 began to increase and the modulus of section 3 began to decrease. The degree of change was growing while the charge increased. There is no obvious change in the modulus of section 2 in the whole process, showing that the change of hydrogel is occurred near the region close to electrode. After the charge quantity is higher than 0.4C, the absolute value of the slope between the modulus of section 1 and 2 is higher than the value between section 2 and 3. Finally, when the charge quantity reached 0.6, the mean value of modulus of section 1 was 1.6 times to original value, and the mean value of modulus of section 3 was 0.6 times to the original stiffness. The mean of compressive modulus reached 32 kPa for section 1 and 13 kPa for section 3. The result shows that the gradient value could be controlled by the value of charge. The charge is affected by the current and time. Therefore, by controlling the current, the stiffness of gradient hydrogel could be time-dependant changed.

**Fig. 4:**
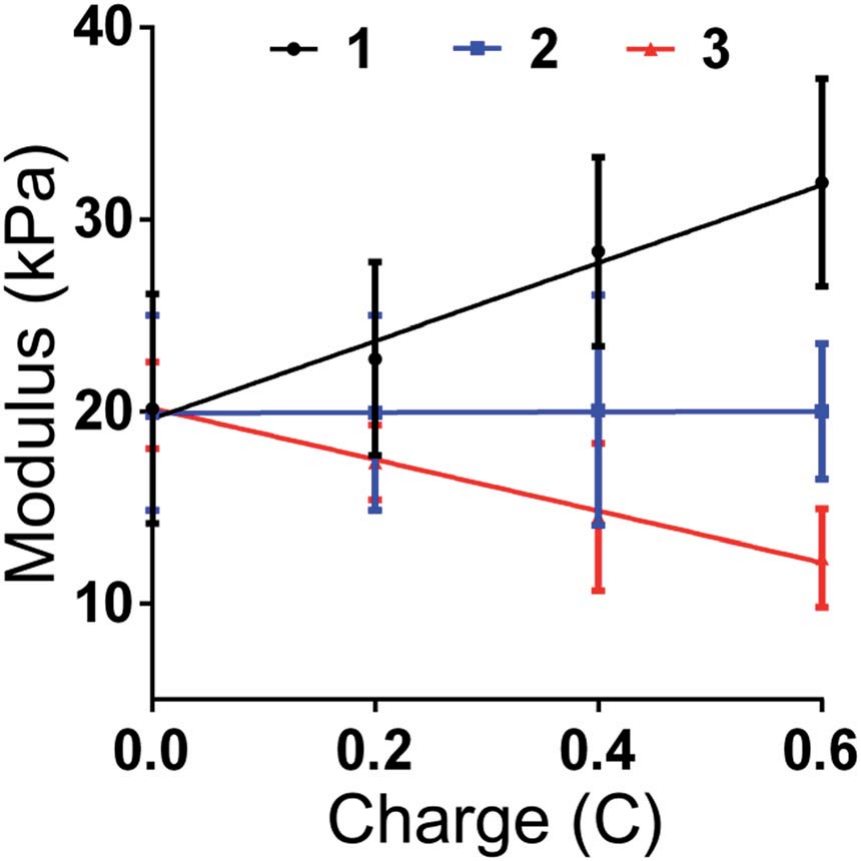
The influence of charge on the modulus of different sections of hydrogel.

An experiment for reversible regulation study was performed, and the he results are shown in Fig. 5 as below. During 0 to 300 s, a voltage (4.5 V) was applied to the hydrogels. The anode was attached to the section 1 while the cathode was attached to the section 3. After 300 s, the electrode was switched. The anode was attached to the section 3 and the cathode was attached to the section 1 from 300 s to 900 s. The modulus *vs.* time was shown in Fig. 5. It is obviously that before 300 s, the change of modulus was as same as pre-mentioned. After the electrode was switched, the modulus of section 1 decreased and the modulus of section 3 increased. The result indicated that the electro-regulation process was reversible.

**Fig. 5:**
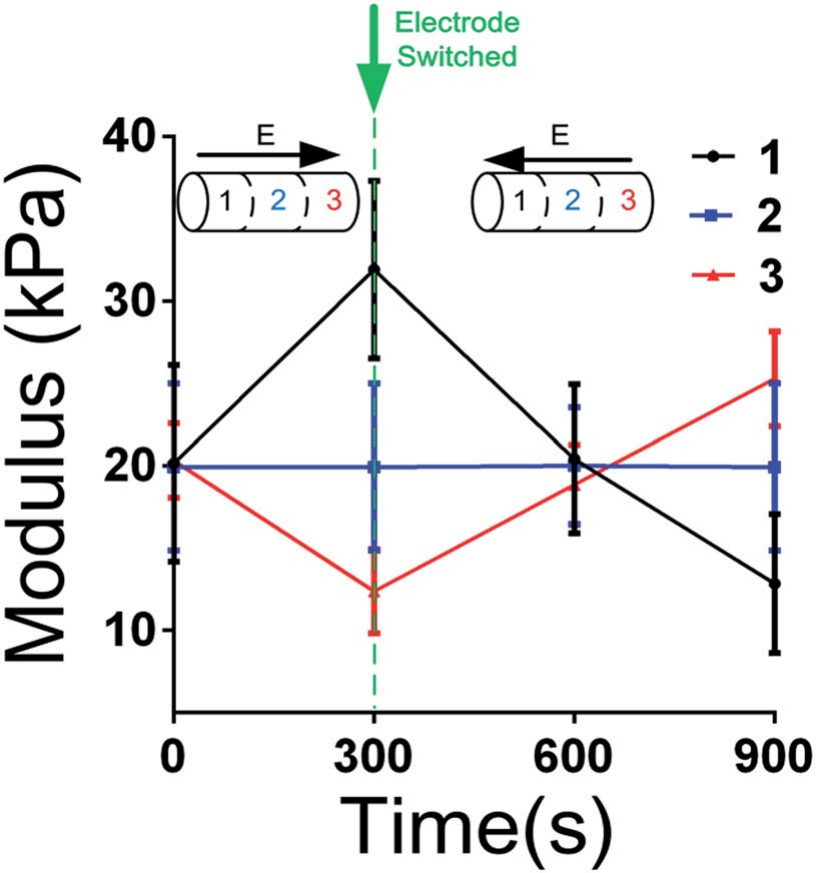
Reversible regulation of the stiffness gradient hydrogel by switching the electrode.

Our results shows that the hydrogels had a gradient stiffness (range from 10 kPa to 30 kPa) which was similar to the modulus in native extracellular matrix, and the stiffness was dynamically regulated by time and electric charge. The process was reversible and controllable. It is reported that a gradient hydrogel (modulus from 1 kPa to 25 kPa) was fabricated by freezing method, however, the modulus could not be dynamically controlled after gelling.^18^ Another gradient hydrogel which was regulated by nanocomposite had the same limitation.^19^ A hydrogel with light-triggered liposomes was developed to overcome this limitation, however, the modulus range of dynamic tuning was below 2 kPa which was lower than the modulus in native extracellular matrix.^20^ Therefore, the importance of our results is that these limitations are overcame by developing a gradient stiffness hydrogel with appropriate modulus and dynamic tuning properties.

## Conclusions

In summary, an electro-regulation method which can dynamically control the stiffness of the hydrogels with ionic crosslinks was proposed. By applying the electric field and controlling the voltage applied to the hydrogel, the mechanical properties of the hydrogel can be time-dependent changed. A gradient stiffness of the hydrogel was generated along the direction of electric field. The initial stiffness of the hydrogel was 20 kPa, and the stiffness could increase to 30 kPa or decrease to 10 kPa in different areas. By controlling the voltage and the duration, the charge can be time-dependent controlled, and any stiffness between 10 kPa and 30 kPa can be dynamically tuned.

## Conflicts of interest

There are no conflicts to declare.

## Supplementary Material

RA-008-C7RA11382J-s001
