# Supplementary material for: Dynamic electro-regulation of the stiffness gradient hydrogels
Source: RSC Adv. 2018 Feb 9;8(12):6675–9. doi: 10.1039/c7ra11382j (PMC9078371; doi:10.1039/c7ra11382j)
Supplement: RA-008-C7RA11382J-s001 [file RA-008-C7RA11382J-s001.pdf]

This journal is © The Royal Society of Chemistry 2018

Time-dependent Stress-relaxation experiments and compressive modulus measurements

The mechanical tests of the electric-induced gradient hydrogel were performed by using a material testing machine which have the compressive strength test mode. The electric-induced gradient hydrogel pieces (diameter 25 mm, thick-ness 8 mm) were placed under the load cylindrical plate. The samples were compressed at a speed of 1 mm/s up to 10% strain, in order to approximate an instantaneous deformation. Within 10% compression, the stresses versus strain relations of the gels are almost linear. Subsequently, the strain was held constant, while the load was recorded as a function of time. The compressive modulus measurements were also performed by the same material testing machine.

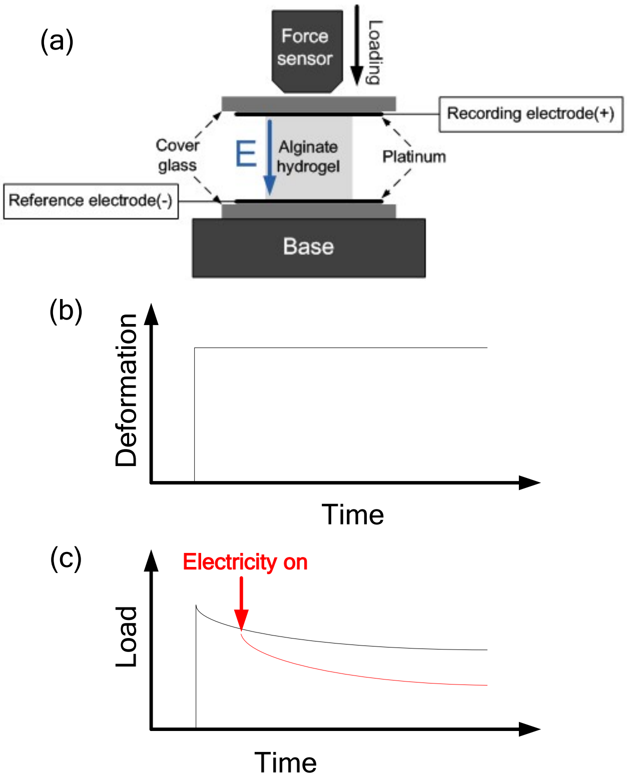

Figure S1. Time-dependent Stress-relaxation experiments.

Preparation of hydrogel

A slow-gelling alginate hydrogel was prepared in this paper. 4% wt sodium alginate was dissolved in deionized water. The calcium ion to carboxyl molar ratio was designed as 0.4. Ca-EDTA was used as a source of calcium ions to initiate gelation. Ca-EDTA to GDL molar ratio of 0.5 was always maintained to achieve a neutral pH value.

Measurement of gradient stiffness properties

The prepared gradient hydrogel (height, 30 mm; diameter, 10 mm) was sectioned along the longitudinal direction by cutting the hydrogel using a blade in order to characterize each section in terms of hydrogel stiffness. The gel was divided into several segments with dimensions of 10 mm height and 10 mm diameter which was cut along the direction of the electric field. The electric field is applied by the electrochemical workstation, and the current was measured simultaneously. The integration of the current is the charge quantity of the hydrogel, which is related to the change of the mechanical property of hydrogels.

Mass loss and charge measurements

It was assumed that the swelling of the gel due to changes in the physicochemistry of bulk

solution would have an influence on solute diffusion. Mass loss was quantified using a mass loss rate, which was determined from the ratio of the mass of the hydrogel before (m_1) and after (m_2) its equilibration in the experimental solutions:

$$S = \frac{m_2 - m_1}{m_1}$$

The charge quantity was calculated by the integration of currents, and the mass loss is recorded by a scales. By the change of the weight, the weight of mass loss was calculated. All measurements were repeated a minimum of three times.

Statistical analysis

The data obtained from each group were averaged and expressed as mean±standard deviation.

Curve fitting for stress-relaxation experiment

The exponential decay equation was used to fit the curve. The exponential decay equation is as follows:

$$y = y_0 e^{-\frac{t}{\tau}}$$

while t is the time taken after the voltage is applied, y0 is the initial value when t equals 0 and τ present the exponential time constant. Especially, τ is the time at which y is reduced to 1/e times its initial value.
